# Automated Detection of Micro-Scale Porosity Defects in Reflective Metal Parts via Deep Learning and Polarization Imaging

**DOI:** 10.3390/nano15110795

**Published:** 2025-05-25

**Authors:** Haozhe Li, Xing Peng, Bo Wang, Feng Shi, Yu Xia, Shucheng Li, Chong Shan, Shiqing Li

**Affiliations:** 1 College of Intelligent Science and Technology, National University of Defense Technology, Changsha 410073, China; lihaozhe21a@nudt.edu.cn (H.L.); wangbonudt@nudt.edu.cn (B.W.); shifeng@nudt.edu.cn (F.S.); xiayu22a@nudt.edu.cn (Y.X.); lishucheng22@nudt.edu.cn (S.L.); 2 National Key Laboratory of Equipment State Sensing and Smart Support, Changsha 410073, China; 3 State Key Laboratory of Functional Crystals and Devices, Shanghai Institute of Ceramics, Chinese Academy of Sciences, Shanghai 201899, China; shanchong@mail.sic.ac.cn; 4 College of Physics, Zhejiang University of Technology, Hangzhou 310023, China; sql@zjut.edu.cn

**Keywords:** additive manufacturing, deep learning, micro–nano defect, defect detection, polarization imaging

## Abstract

Aiming at the key technology of defect detection in precision additive manufacturing of highly reflective metal materials, this study proposes an enhanced SCK-YOLOV5 framework, which combines polarization imaging and deep learning methods to significantly improve the intelligent identification ability of small metal micro and nano defects. This framework introduces the SNWD (Selective Network with attention for Defect and Weathering Degradation) Loss function, which combines the SIOU Angle Loss with the NWD distribution sensing characteristics. It is specially designed for automatic positioning and identification of micrometer hole defects. At the same time, we employ global space construction with a dual-attention mechanism and multi-scale feature refining technique with selection kernel convolution to extract multi-scale defect information from highly reflective surfaces stably. Combined with the polarization imaging preprocessing and the comparison of enhancement defects under high reflectivity, the experimental results show that the proposed method significantly improves the precision, recall rate, and *mAP*50 index compared with the YOLOv5 baseline (increased by 0.5%, 1.2%, and 1.8%, respectively). It is the first time that this improvement has been achieved among the existing methods based on the YOLO framework. It creates a new paradigm for intelligent defect detection in additive manufacturing of high-precision metal materials and provides more reliable technical support for quality control in industrial manufacturing.

## 1. Introduction

The service performance of high-end equipment, such as aerospace, medical devices, and military equipment, depends largely on the performance of its components. Most components are used in extremely harsh environments; they are required to have strong load-bearing, extreme heat resistance, light weight, strong corrosion resistance, and high reliability. This poses a grave challenge to the components’ material, structure, process, and performance. Metal additive manufacturing (AM), also known as 3D printing, is a technique in which metal powders are formed by stacking them layer by layer and melting and bonding them, which has revolutionized the processing mode of traditional metal construction. This mainly includes several common processes, such as selective laser melting (SLM), electron beam melting (EBM), and powder bed fusion (PBF). Among them, laser selective melting (LSM) technology is one of the most popular and common research fields in AM technology. It uses a high-energy laser [1] to focus on a thin layer of metal powder, precisely melting the powder and solidifying it into a layer. Then, it moves to the next layer to continue this process, which is suitable for manufacturing complex shapes and high-strength materials. Compared with the traditional metal processing mode, AM technology has high production flexibility, no need for conventional molds, fast manufacturing speed, the ability to manufacture complex geometric structures, and reduced material waste. With the advancement of technology, metal additive manufacturing technology has become an essential part of modern manufacturing. However, in its high-quality production processes, due to severe process instability, it is prone to defects such as pores, poor fusion, spheroidization, cracks, and surface roughness, resulting in scrapping of the workpiece. Quality control in an efficient production mode has become a key bottleneck because even a minor defect can lead to the failure of the entire product. Therefore, studying the intelligent defect detection algorithm of metal additive technology is of great significance.

Traditional defect detection techniques, such as optical microscopy or ultrasonic flaw detection, are often limited by the experience and skill level of the operator, making it challenging to automate inspections on a large scale quickly, as these techniques are prone to missed detections. To detect the generation of defects in real time and eliminate defective products promptly, the development of machine learning and deep neural networks provides new solutions for defect detection and classification. In advanced computer vision, the defect detection task is mainly optimized through two primary strategies—segment-based and integrated methods. Segment-based models include RCNN, Fast R-CNN, and Faster R-CNN [2]. Based on the prior region proposal technology, features are extracted through convolutional neural networks (CNN) [3,4,5], and then, each candidate region is finely classified, and its bounding box is adjusted separately. Although these studies provide high recognition accuracy, they do not have real-time recognition capabilities. However, for example, in the SSD (Single Shot multibox Detector) and YOLO (You Only Look Once) series, to balance efficiency and recognition accuracy, these models avoid the generation of candidate regions and directly distinguish the location and category of the predicted target in the whole image, which gives them a significant advantage in real-time identification of large-scale targets. In recent years, especially in convolutional neural network (CNN)-based object detection frameworks such as the YOLO series, machine learning algorithms have been widely used in defect detection, but due to the inaccurate image accuracy recognition of low-resolution and small targets, it is necessary to introduce new intelligent algorithms.

The YOLO series of object detection algorithms relies on their excellent detection speed and accuracy in defect detection [6]. As Li et al. [7] pointed out, although these technologies have made breakthroughs in improving the accuracy of defect detection, they still fall short when facing specific difficulties. Due to the lack of detailed information, CNNs may not be able to accurately identify potential defects in low-resolution scan results [8]. Complex background interference often makes defect detection more difficult. The network needs stronger background noise suppression [9], especially for small-sized targets, notably for defects with similar shapes but significant size differences, and existing models may be misjudged or missed. To overcome these challenges, researchers are constantly innovating technologies, such as combining multi-scale feature fusion, which can capture defect information at different scales [10], and introducing attention mechanisms to enhance the network’s attention to critical regions. Li et al. [11] proposed a steel defect detection algorithm based on the improved YOLO, which consists of 27 convolutional layers and improves the detection accuracy. Liang et al. [12] proposed an improved YOLOv3-Tiny model, using K-means to enhance the regression accuracy and improve the network’s ability to extract feature information. Yu et al. [13] improved the backbone network, embedding a fusion attention mechanism module and focusing more on channel and spatial data by introducing the Focal Loss function to solve the problem of uneven sample distribution. They proposed an improved algorithm of YOLOv4. Kwon H et al. [14] presented a new QSAR model using semi-supervised metric learning techniques to assess which chemical functional groups affect bioactivities toward specific biological targets, Huang et al. [15], to enhance the detection accuracy of the voids in laser-cladding damage-repair parts, creatively proposed the SP-YOLOv5 void detection algorithm. This research added the Coord attention mechanism module, rewrote the YOLOv5 network structure, and enhanced the network model’s detection ability for small target voids of the class. Bhagabati et al. [16] proposed an improved detection algorithm based on YOLOv5s. This research added a multi-scale attention mechanism module and designed a recursive gated convolutional C3HNB module to address the complex spatial information of small target detection images. Lin et al. [17] utilized mixed attention to optimize the detection of small targets in SAR images. This research combined channel attention, self-attention mechanism, and context feature fusion strategy to enhance detection performance. Shiga M et al. [18] presented a novel approach for the classification and visualization of rock microstructure from micro-computed tomography images, leveraging pre-trained convolutional neural network (CNN) models (AlexNet, GoogLeNet, Inception v3 Net, ResNet, and DenseNet) combined with unsupervised machine learning (USML) techniques principal component analysis, multidimensional scaling, isometric mapping, t-distributed stochastic neighbor embedding (t-SNE), and uniform manifold approximation projection (UMAP)). Huang et al. [19] designed the C3-ODCBS module, introducing the WIoUv3-Loss function and decoupled heads, enabling the model to exhibit higher detection accuracy on steel defects. Bearce et al. [20], based on the improved YOLOv8n target lightweight detection algorithm DCD-YOLO, utilized the CDR module on the backbone network of YOLOv8 for feature extraction and added the bidirectional feature pyramid BiFPN at the neck to achieve more rapid and efficient multi-scale feature fusion, thereby enhancing its detection capability. Shin et al. [21] made improvements based on YOLOv10 by introducing the attention mechanism EMA and using the SPPELAN module. They replaced the backbone with FasterNet. The study improved the detection accuracy, training efficiency, and complex defect recognition ability; the computational complexity was also kept low, ensuring the deployability and usefulness of the model in resource-constrained environments.

In summary, machine learning has achieved a certain degree of success in additive manufacturing defect detection; it has shown specific value and efficiency in existing application scenarios. There is still much room for improvement in the performance of known algorithms. Among them, the two key problems of defect detection in complex scenes and small target defect detection are particularly prominent, which are the most significant problems hindering the detection accuracy of the algorithm. There is an urgent need for innovative solutions to achieve substantial breakthroughs [22], and the core highlight and major innovation of this research lies precisely in the fact that the research successfully overcomes the problems of defect detection in complex scenarios and small targets. A more systematic solution is thus proposed, effectively improving the current algorithm’s shortcomings in these aspects. The further expansion of the results of this study is expected to promote the overall leapfrog upgrade of the additive manufacturing industry at the two levels of quality control and production efficiency. It will build a solid theoretical foundation and technical support framework for the long-term and sustainable development of the field of additive manufacturing, lead the field to a new height of development, and stimulate more innovative applications and research explorations.

In this study, to further enhance the detection accuracy of defects, the network structure of YOLOv5 was modified based on YOLOv5s. (1) The SiOULoss function and the NWDLoss function were fused to creatively propose a new loss function, SNWDLoss, which accelerates the network convergence and improves the robustness. (2) The CA and SK attention mechanisms complement each other’s shortcomings and were combined into the bottleneck to effectively enhance the algorithm’s attention to local features and its ability to organize global sequence information.

The main contributions of this paper are as follows:

(1) The CA attention mechanism module is designed to be highly flexible and resource-efficient. In this paper, the CA attention mechanism is seamlessly integrated into the bottleneck in the core architecture of the mobile network. The position information of channel attention and direction perception is cleverly integrated, which significantly improves the model’s accuracy in localization and recognition and shows excellent versatility and performance improvement in diverse tasks such as defect detection. To improve the computational efficiency of the model, this study introduced the SK attention mechanism into the bottleneck, focusing on the key parts of the task in the input feature map, better preserving the spatial structure of the image, and enhancing the adaptability and generalization of the model to various scenes. At the same time, the SK attention mechanism can be targeted at specific areas, such as defects, which can reduce the risk of overfitting.
(2) CioULoss was replaced with SIoULoss, a loss function in semi-supervised learning. It replaces the traditional CIoU Loss and guides the model to learn more representative features from only the model when the dataset of metal defects is relatively small. Meanwhile, the NWDLoss function is introduced into SIoULoss to consider the influence of noisy data and dynamically adjust the weight of each sample. This mechanism can adaptively handle the noise points in the defect dataset and reduce the impact of noise on model training.
(3) To improve the accuracy and production efficiency of defect detection in additive manufacturing parts, a novel defect identification network structure, SCK-YOLOv5, was innovatively designed. It is optimized on the classic YOLOV5 architecture, adding CA and SK attention mechanisms. Positioning is more accurate using a modified version of SIoULoss instead of the traditional CIoULoss. The SNWDLoss loss function model is innovatively proposed, combining the two loss functions of SIOULoss and NWDLoss to effectively improve the overall detection performance, significantly improving the model’s effectiveness in practical applications.
(4) In this study, the innovative combination of polarization imaging and deep learning algorithms was used to highlight the defects of small targets, highlighted by the temperature of the metal surface and the phenomenon of high reflection, achieving high-precision detection of small target defects. This improves the detection accuracy and lays a solid foundation for quality control and safety assurance in related fields.

## 2. Methodology

### 2.1. Polarized Defect Detection System

The inspection system includes illumination, optics, sensors, and signal processing units, and the selection of the appropriate imaging method will significantly impact the detection efficiency and identification accuracy of defects. In the defect detection process, different temperature conditions, uneven light distribution, and highlight areas generated by the reflection of metal surfaces will mask the defect’s important feature information, making it difficult to accurately reflect its contour and morphological information. Conventional optical inspection methods (such as RGB imaging) have significant limitations in metal surface inspection: (1) high-reflective areas can lead to local overexposure or specular reflection noise, masking the features of minute defects; (2) anisotropic scattered light interference can reduce the signal-to-noise ratio, but the polarization imaging is not affected by the surface temperature or high reflectivity of the defective target, which reduces the interference of background clutter, effectively highlights the defects in the background, and solves the problem of masking defects in high-reflectivity areas to prevent the geometric texture information from being accurately restored.

**Figure 1:**
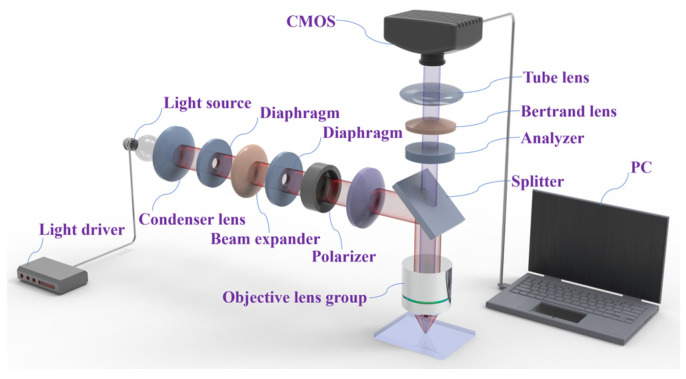
The schematic diagram of the polarized defect detection system.

To effectively address these challenges, we propose a polarization defect detection system based on polarization machine vision and deep learning; Figure 1 shows a schematic diagram of the proposed defect detection system setup, which has two main components: a polarization imaging subsystem and an automated deep learning processing subsystem. The polarized imaging subsystem contains an objective lens group, a light source, a Bertrand lens, a tube lens, a condenser lens, a diaphragm, a beam expander, a polarizer, an analyzer, and a high-resolution CMOS sensor. The light source is a 12 V 30 W halogen lamp with adjustable brightness for different materials. Both the polarizer and analyzer can rotate 360°. The objective lens group has a dual telecentric achromatic structure. Its working distance is about 20 mm, the numerical aperture is 0.25, and the magnification is 10 times. The CMOS sensor has a pixel size of 4.54 microns and a chip size of 1 inch. The focal length of the tube lens is 200 mm. The polarizer has four positioning angles of 0, 90, 270, and 360. At 0°, the polarization direction is positive east–west, and the analyzer can rotate 360° with a minimum positioning accuracy of 0.1°. The minimum aperture of a variable diaphragm is 1.5, and the maximum aperture is 22. The automatic deep learning processing subsystem mainly deals with data augmentation, defect identification, and classification.

### 2.2. Detection Process

**Figure 2:**
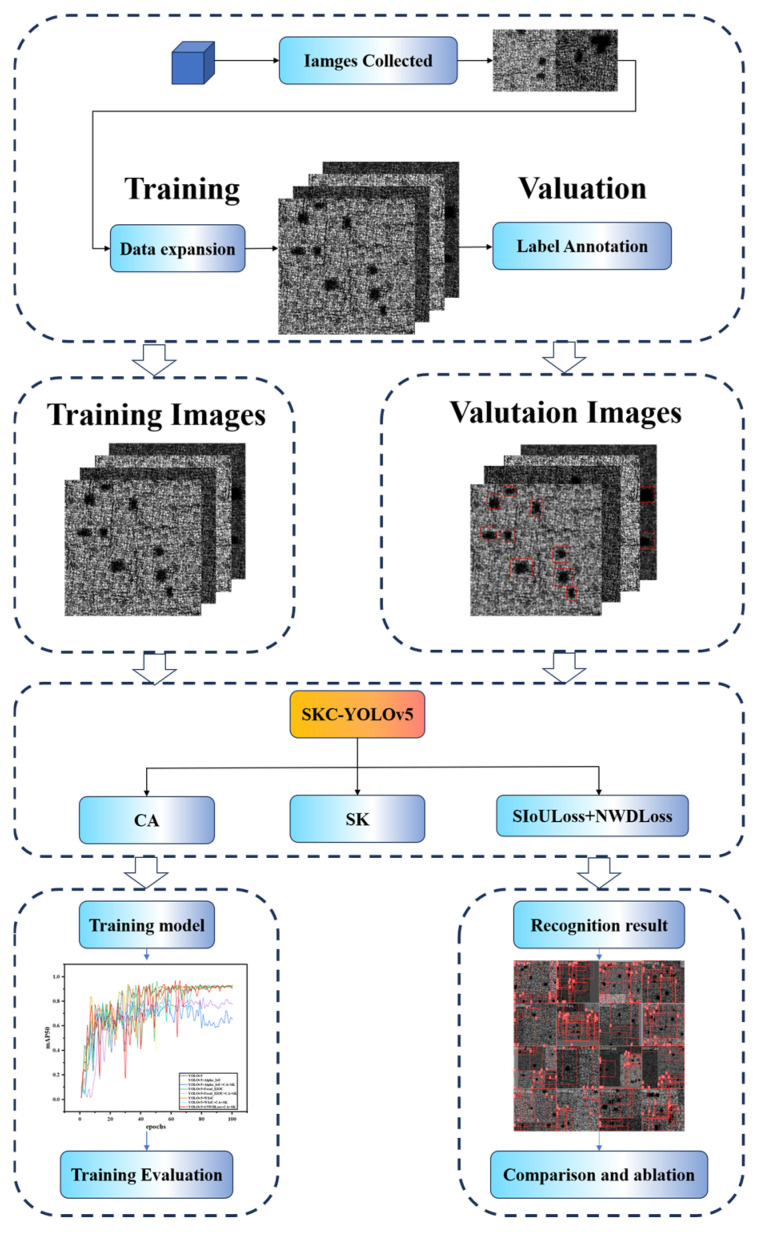
Flow chart of SCK-YOLOV5 defect detection.

Figure 2 shows the defect detection process flowchart of SCK-YOLOv5, covering four main stages: dataset extraction, batch image processing, model training, and performance evaluation. Based on the polarized defect detection system, a small number of defects on the laser additive manufacturing workpiece were extracted and photographed. The extracted images were processed, and the position and category information of the processed images were marked with Label1Img and saved in a text file. The number of datasets extracted in this way was limited, so we cooperated with Beijing University of Technology to enhance the data, which helped improve the model’s generalization ability. Due to the limited number of datasets, after operations such as inversion and rotation, combined with the existing image expansion techniques, we only obtained a dataset of 1000 metal defects with holes. This paper conducts training based on this; this may impact the identification of other types of datasets, resulting in inaccurate detection accuracy. In the training phase, we designed the SCK-YOLOv5 model to improve training accuracy. The model combines the CA attention mechanism, the SK attention mechanism, the SIoULoss function, and the NWDLoss function. Regarding the evaluation phase, in this study, we made sure to use the same set of tests, and we also established a set of performance evaluation metrics that included precision, recall, mean average precision (*mAP*@50, *mAP*@50–95), regression value, and several key parameters. Then, the detection performance of different models was analyzed, and the defect detection algorithms based on deep learning were compared to verify the effectiveness of the defect detection model proposed in this paper.

### 2.3. The Principle of the SCK-YOLOv5 Defect Detection Model

#### 2.3.1. YOLOv5 Model

**Figure 3:**
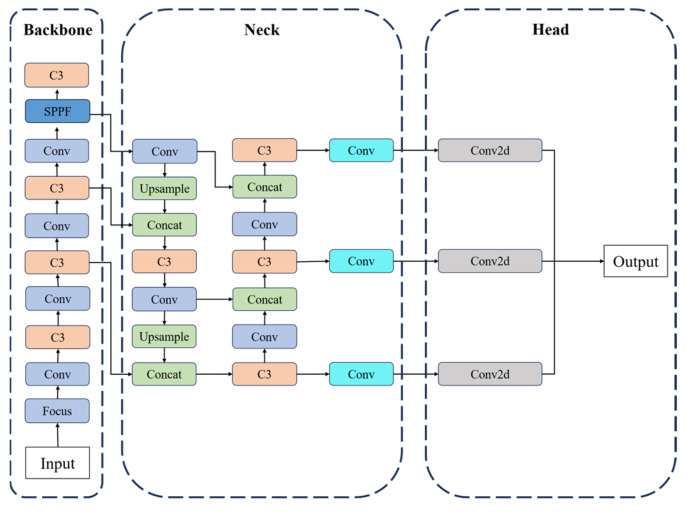
Network structure diagram of YOLOv5.

YOLOv5 is an efficient single-stage object detection algorithm that simplifies the process by directly predicting the position and category of each candidate box [23]. Figure 3 shows the structure of the original YOLOv5 network, with the backbone consisting of multiple convolutional blocks, such as Conv, C3, SPPF, etc., which is the central part of the neural network and is responsible for extracting features from the original input data.

#### 2.3.2. Improvement of YOLOv5 Model

**Figure 4:**
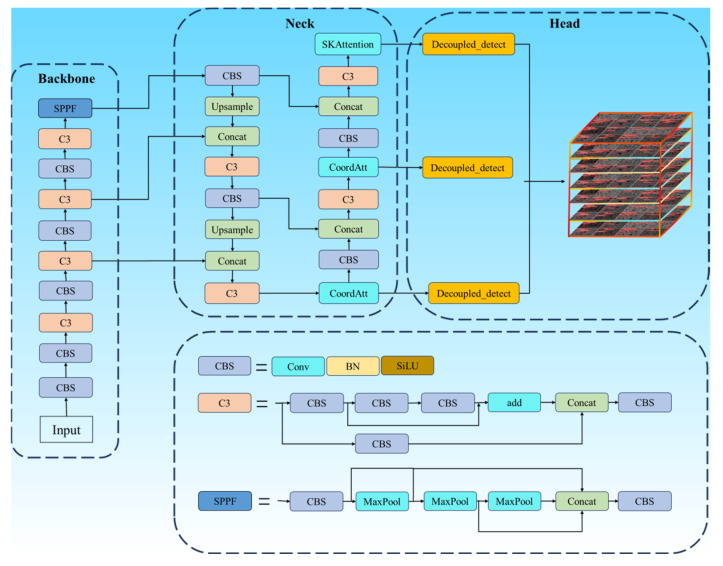
Schematic diagram of the structure of the SCK-YOLOv5 model.

**Figure 5:**
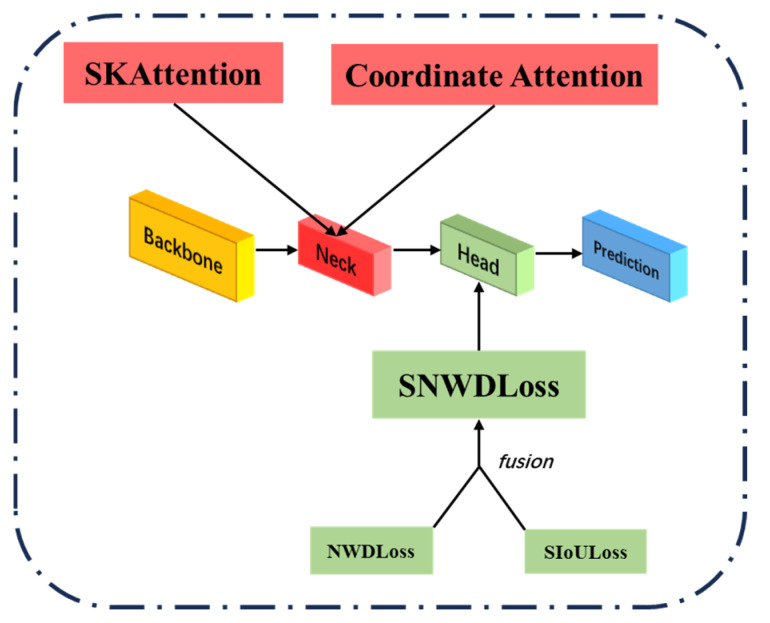
Schematic diagram of the improved core of SCK-YOLOv5.

(1) To enhance the practical performance of YOLOv5 in the defect detection task of additive manufacturing, this study made two major improvements to YOLOv5 and established an additive manufacturing defect detection model, SCK-YOLOv5, based on the improved YOLOv5, as shown in Figure 4. To improve the model performance, this study adopted SIOU instead of the traditional CIOU loss function, which accelerated the training process and enhanced the model’s accuracy and anti-interference ability. This study was further innovated by introducing NWDLoss and constructing a novel SNWDLoss function framework. As shown in Figure 5, this loss function based on Wasserstein distance essentially measures the “migration cost” between two predictions and the true distribution, particularly enhancing the model’s perception of small sample variations and significantly improving the stability and consistency in similar sample recognition [24].

(2) In the model design, this study integrates the CA (Coordinate Attention) mechanism, which not only focuses on the interaction between channels but also considers the directionality of positional information and is endowed with both flexibility and efficiency. It can seamlessly integrate into the key modules of lightweight networks [25]. CA focuses on the spatial and channel dimensions and overcomes the problem of long-distance dependence. In practical applications, CA tries to maintain as high an accuracy as possible while effectively controlling the number of parameters and computational load.

(3) SK-Attention (Selective Convolution Kernel Attention) was integrated as a selectivity mechanism utilizing the dynamic size of convolutional kernels. The essence of this mechanism is to allow neurons to flexibly and automatically adjust their feelings based on multiscale inputs. By doing so, it boosts the network’s capacity to handle multiscale features, as documented in reference [26]. This attention mechanism does not just enhance the model’s performance. It also demonstrates outstanding efficiency traits, showing great promise in optimizing the model’s operation.

This study improved the performance of the YOLOv5 model in incremental manufacturing defect detection through several improvements. SIOU replaced the CIOU loss function to accelerate the training and improve the accuracy. The NWDLoss framework was introduced to enhance the stability of small sample variation perception and similar sample identification. The CA mechanism was integrated to improve the flexibility and efficiency of the model and, at the same time, solving the long-distance dependence problem. The SK-Attention mechanism was introduced to optimize the multi-scale feature sensitivity and significantly reduce the computational burden. These improvements in YOLOv5 show stronger performance and practical application value in incremental manufacturing defect detection.

#### 2.3.3. SK-Attention Mechanism

**Figure 6:**
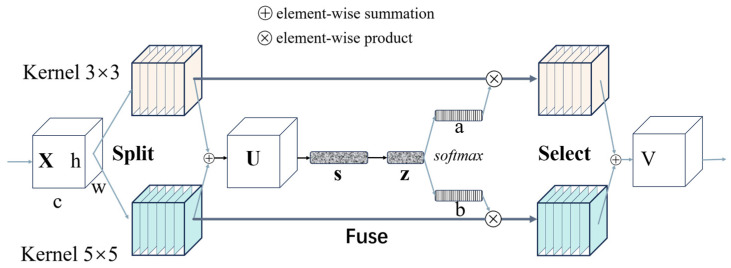
Schematic diagram of SK-Net structure.

The SK-Attention mechanism enables the network to capture multi-scale features more effectively in the image space while reducing computing resource waste [27]. SK-Net [26] includes three operations, namely Split, Fuse, and Select, to realize the fusion and selection of multi-scale information. Figure 6 shows the specific structure diagram of SK-Net.

It can be seen that the Split operation first processes the input features through convolution kernels of different sizes to capture multi-scale feature information; then, these different scales are concatenated together and enter the Fuse operation for global pooling, dimensionality reduction, dimensionality increase, and softmax normalization processing to generate weights of various scales; finally, the Select operation selects the most effective feature scale by weighted summation of the weights of each scale and the convolution output from the Split operation, thereby achieving focused detection [26].

#### 2.3.4. CA Mechanism

**Figure 7:**
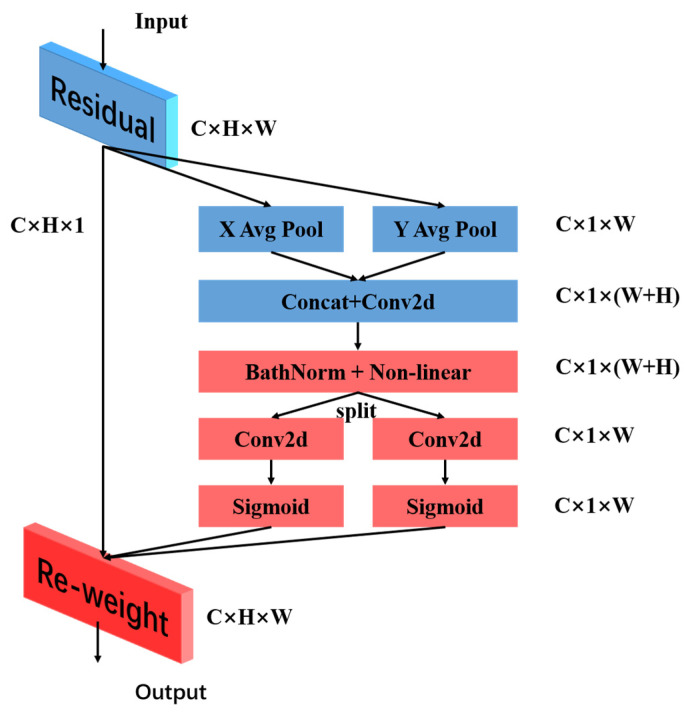
Schematic diagram of the structure of the attention mechanism of CA.

Coordinate Attention can enable modeling operations for channel correlations and long-term dependencies. As shown in Figure 7, this mechanism mainly consists of two major steps: embedding coordinate information and generating coordinate attention. In the coordinate information embedding stage, specifically, location-related information is embedded into the input feature map. An attention map is constructed in the coordinate attention generation stage based on the existing location information. Then, this attention is applied to the input feature map, highlighting representative contents that deserve attention. The advantage of Coordinate Attention [28] is that it can model large areas and effectively avoid large-scale computational costs when in a mobile network environment.

#### 2.3.5. SIoULoss Function

In machine learning, the loss function is like a “referee” determining whether the model’s predicted outcome is accurate compared to the desired result. In object detection, the YOLOv5 model uses the CIoU loss function. The model gives a prediction box and a real box. CIoU is a loss function that considers two aspects to evaluate the accuracy of a prediction. The closer the distance between the prediction box and the real box, the better; regarding the size of the overlapping area of the two boxes, the more overlapping the area, the more accurate the prediction. Combining these factors allows CIoU to determine better whether the YOLOv5 model predictions are correct. The penalty metric of CIoU is based on the DIoU penalty metric. It adds factor α v, which considers the matching situation of the aspect ratios of the predicted and real boxes.

The formula for the penalty term is as follows [29]:

(1) $$\;R_{CIoU}=\frac{\rho^{2}\left(b,b^{gt}\right)}{c^{2}}+v$$

The definition of α is as follows:

(2) $$\;\alpha =\frac{v}{\left(1-IOU\right)+v}$$

It is a parameter used to measure the consistency of aspect ratio, and v is defined as follows:

(3) $$\;\begin{array}{c} v=\frac{4}{\pi^{2}}{\left(\arctan\frac{w^{gt}}{h^{gt}}-\arctan\frac{w}{h}\right)}^{2} \end{array}$$

The formula of the complete *CIoU* loss function is as follows:

(4) $$\;L_{CIoU}=1-IOU+\frac{\rho^{2}\left(b,b^{gt}\right)}{c^{2}}+\alpha v$$

However, *CIoU* [29] has a defect because it does not consider the mismatch directions between the required ground truth boxes and the predicted “experimental” boxes. This deficiency slows down the convergence rate and reduces the efficiency. The reason is that the predicted boxes may encounter situations such as oscillation and divergence during training, leading to a deterioration in model performance. Therefore, this study replaced the loss function *CIoU* in YOLOv5 with SIoU, which fully considers the vector angles between the required regressions and redefines the penalty indicators. They are angle, distance, shape, and *IoU* costs. The calculation formulas are as follows:

Angle cost (Λ):

(5) $$\;\Lambda =1-2\times \sin^{2}\left(\arcsin\left(x\right)-\frac{\pi }{4}\right)$$

The angle difference between the line connecting the centers of the predicted bounding box and the ground truth (GT) bounding box and the horizontal axis, x, is the value of $\sin\left(\alpha \right)$, when $\arcsin\left(x\right)=\frac{\pi }{4}$,  Λ =1; when $\arcsin\left(x\right)=0$,  Λ = 0;

Distance cost:

(6) $$\;\Delta =\sum_{t=x,y}\;\left(1-e^{-\gamma \rho_{t}}\right)$$

(7) $$\;\rho_{x}={\left(\frac{b_{cx}^{gt}-b_{cx}}{c_{W}}\right)}^{2},\rho_{y}={\left(\frac{b_{cy}^{ty}-b_{cy}}{c_{h}}\right)}^{2},\gamma =2-\Lambda$$

The normalized center point distance is adaptively adjusted according to the angle difference. $c_{W}$ and $c_{h}$ are the widths and heights of the smallest enclosed rectangle containing the predicted and real boxes, respectively. The $b_{cx}^{gt}-b_{cx}$, $b_{cy}^{ty}-b_{cy}\;$ calculation is the difference between the center position of the real box and the predicted box on the x, y-axis. The purpose of dividing by $c_{W},\;\;c_{h},$ is to normalize this difference so that it is within a certain range so as to avoid the distance difference between the boxes at different scales from being too large and affecting the loss calculation. The last square is taken to ensure that the result is non-negative and to increase the penalty when the distance difference is large. Δ is the angle loss calculated earlier and is used to measure the difference in angle between the predicted and true boxes. γ is a coefficient related to angular loss, which is used to adjust the weight of distance loss according to the magnitude of the angular difference.

$1-e^{-\gamma \rho_{t}}\;$ is the formula for calculating the final distance loss. When the distance between the prediction and true boxes on the x-axis and y-axis is small, the values of both exponential functions are close to 1, and the distance loss is close to 0, and when the distance difference is large, the values of both exponential functions are close to 0, and the distance loss is close to 2. This effectively measures the difference in the spatial position of the prediction box and the true box and gives a larger penalty when the distance difference is large.

Shape cost:

(8) $$\;\Omega =\sum_{t=w,h}\;(1-e^{-\omega_{t}}{)}^{\theta }$$

Considering the relative difference in aspect ratio, the value of the exponential function is close to 1 when $\omega_{w}$ or $\omega_{h}$ is small, that is, when the difference in width or height between the predicted and true boxes is small, and close to 0 when $\omega_{w}$ or $\omega_{h}$ is large. Using $1-e^{-\omega_{t}}$, the purpose of this step is to invert the value of the exponential function. A fourth-power operation is performed on the above results. Power operations increase the penalty when there is a large difference. Finally, the shape loss of width and height is added together to obtain the final shape loss shape_cost. This allows for a combination of the difference in shape in width and height between the predicted and real boxes.

*IoU* cost:

(9) $$L_{box}=1-IoU+\frac{\Delta +\Omega }{2}$$

*Iou* is the intersection and union ratio of the prediction box and the real box, and the final SIoULoss is obtained by subtracting the weighted sum of distance loss and shape loss from the intersection union ratio. Here, the weight of both the distance loss and the shape loss is 0.5.

#### 2.3.6. NWDLoss Function

NWDLoss is a loss function in deep learning, specifically designed for image generation tasks. Its underlying logic is based on the Wasserstein distance, a metric that measures the “distance” between two probability distributions. The main idea is to see the goal of the generative model as a process of making the distribution of the generated image as close as possible to the distribution of the real data, which influences neurons. Compared with traditional loss functions such as MSE (mean square error) or KL divergence, the Wasserstein distance emphasizes the overall structural consistency and detail accuracy of the generated image, and the model strives to reduce the “transmission cost” between the generated image and the actual real image in the calculation. NWDLoss optimizes the dual-input network architecture, where one generator is designed to create images similar to training examples. At the same time, the other discriminator evaluates the difference between the two, the generator updates the parameters to minimize the predictive value of the discriminator, and the discriminator works on the prediction maximization by analyzing different images so that its prediction is better. The generators are driven to gradually align with the real data distribution through this competing process.

For the small target defect detection in our study, we use the Wasserstein distance to measure the similarity of the bounding box more accurately. Therefore, we introduce the NWDLoss function, which adopts an innovative framework that treats each bounding box as a two-probability density function, essentially a two-Gaussian distribution. The method can quantify and evaluate their similarity by calculating the normalized Wasserstein distance between these two Gaussian distributions (normalized Wasserstein distance). This comparison strategy based on high-dimensional probability distribution is particularly effective in accurately identifying subtle changes in small targets, thus significantly improving the accuracy of defect detection, improving the adaptability to complex scenarios, and enhancing the robustness of the whole system. Thus, we model the bounding box as a 2D Gaussian distribution.

The equation of the inscribed ellipse is as follows:

(10) $$\;\begin{array}{c} \frac{(x-\mu_{x}{)}^{2}}{\sigma_{x}^{2}}+\frac{(y-\mu_{y}{)}^{2}}{\sigma_{y}^{2}}=1 \end{array}$$

For the horizontal bounding box, R = ($c_{x}$, $c_{y}$, *w*, *h*), where ($c_{x}$, $c_{y}$), *w* and *h*, respectively, represent the center coordinates, width, and height; ($\mu_{x},\;\mu_{y}$) represents the center coordinates of the ellipse; $\sigma_{x}\;\;and\;\sigma_{y}$ are the half-axis lengths along the x-axis and y-axis, respectively. $c_{x}\;$=$\;\mu_{x},\;$*c_y_* =$\;\mu_{y}$, $\sigma_{x}\;$=$\;w^{2}$, and $\sigma_{y}\;$=${\;h}^{2}$.

The probability density function of the two-dimensional Gaussian distribution is given by the following, where *x*, μ, and Σ represent the Gaussian coordinates (*x*, *y*), the mean vector, and the covariance matrix, respectively:

(11) $$\;f\left(x|\mu ,\;\Sigma \right)=\frac{exp(-\frac{1}{2}(x-\mu {)}^{⊺}\Sigma^{-1}(x-\mu ))}{2\pi |\Sigma {|}^{\frac{1}{2}}}$$

This formula describes the probability density function of a two-dimensional Gaussian distribution. The exponential part $-\frac{1}{2}(x-\mu {)}^{⊺}\Sigma^{-1}(x-\mu )$ measures the “distance” of point x from the mean μ, and this distance is scaled by the covariance matrix Σ. The denominator $2\pi |\Sigma {|}^{\frac{1}{2}}$ is a normalization constant that ensures the integral of the entire probability density function over the entire two-dimensional plane equals 1.

(12) $$\;{\left(x-\mu \right)}^{⊺}\Sigma^{\left(-1\right)}\left(x-\mu \right)=1$$

When $\;{\left(x-\mu \right)}^{⊺}\Sigma^{\left(-1\right)}\left(x-\mu \right)=1$, all the points x that satisfy this equation form an ellipse, and this ellipse is the density contour of a two-dimensional Gaussian distribution. The center of this ellipse is the mean vector μ, and its shape and direction are determined by the covariance matrix Σ.

The inner-enclosed ellipse will be the density contour of a two-dimensional Gaussian distribution. Therefore, the two-dimensional Gaussian distribution N**(**μ**,** Σ**)** can be modeled by the horizontal bounding box R = ($c_{x}$, $c_{y}$, *w*, *h*).

(13) $$\;\mu =\left[\begin{array}{c} c_{x}\\ c_{y} \end{array}\right],\Sigma =\left[\begin{array}{cc} \frac{w^{2}}{4} & 0\\ 0 & \frac{h^{2}}{4} \end{array}\right]$$

A horizontal bounding box is used to approximately represent the two-dimensional Gaussian distribution. The two components of the mean vector μ are the center coordinates $c_{x}$ and $c_{y}$ of the bounding box. The covariance matrix Σ is a diagonal matrix, with the diagonal elements being $\frac{w^{2}}{4}$ and $\frac{h^{2}}{4}$, which indicates that the two dimensions are independent of each other, and the width and height of the distribution are determined by the width and height of the bounding box, respectively.

The Wasserstein distance is adopted as the most optimal metric to calculate the distribution distance. For $\mu_{1}\;$= N ($m_{1}$, $\Sigma_{1}$) and $\mu_{2}\;$= N ($m_{2}$, $\Sigma_{2}$), the second-order Wasserstein distance between $\mu_{1}$ and $\mu_{2}$ is defined as follows:

(14) $$\;W_{2}^{2}\left(\mu_{1},\mu_{2}\right)={∥m_{1}-m_{2}∥}_{2}^{2}+Tr\left(\Sigma_{1}+\Sigma_{2}-2{\left(\Sigma_{2}^{\frac{1}{2}}\Sigma_{1}\Sigma_{2}^{\frac{1}{2}}\right)}^{\frac{1}{2}}\right)$$

This second-order Wasserstein distance is used to measure the difference between two Gaussian distributions. It consists of two parts: the first part is the square of the Euclidean distance between the two mean vectors, which measures the difference between the centers of the two distributions; the second part is a term involving the covariance matrix, which measures the differences in the shapes and directions of the two distributions.

It can be simplified:

(15) $$W_{2}^{2}\left(\mu_{1},\mu_{2}\right)={∥m_{1}-m_{2}∥}_{2}^{2}+{∥\Sigma_{1}^{\frac{1}{2}}-\Sigma_{2}^{\frac{1}{2}}∥}_{F}^{2}.$$

For the Gaussian distributions $N_{a}\;\;and\;N_{b}$ modeled by bounding boxes A = ($cx_{a}$, $cy_{a}$, $w_{a}$, and $h_{a}$) and B = ($cx_{b}$, $cy_{b}$, $w_{b}$, and $h_{b}$), the equation can be further simplified as follows:

(16) $$\;W_{2}^{2}\left(N_{a},N_{b}\right)={∥\left({\left[cx_{a},cy_{a},\frac{w_{a}}{2},\frac{h_{a}}{2}\right]}^{T},{\left[cx_{b},cy_{b},\frac{w_{b}}{2}.\frac{h_{b}}{2}\right]}^{T}\right)∥}_{2}^{2}.$$

When two Gaussian distributions are represented by bounding boxes, the second-order Wasserstein distance can be further simplified to the square of the Euclidean distance of a vector containing the parameters of the bounding boxes.

By using its exponential form for normalization, a new metric called normalized Wasserstein distance (NWD) is obtained [30]:

(17) $$NWD\left(N_{a},N_{b}\right)=\exp\left(-\frac{\sqrt{W_{2}^{2}\left(N_{a},N_{b}\right)}}{C}\right)$$

By taking the square root of the second-order Wasserstein distance and dividing it by a constant C and then taking the negative exponent, the normalized Wasserstein distance is obtained. This distance value lies between 0 and 1. The larger the value, the more similar the two distributions are.

#### 2.3.7. Improvement and Interaction of the Components

This study is based on the YOLOv5 model and successively introduces the CA attention mechanism and the SK attention mechanism in its neck. The interaction between the two first generate the advantage of sequential complementarity. The CA attention mechanism first extracts direction-sensitive position information, enhancing the perception ability of the feature image to position information. Subsequently, based on these improved characteristics, the SK attention mechanism further enhances the feature expression through multi-scale convolution and dynamic selection to capture targets of different sizes and shapes. Secondly, the complementarity of the two will lead to the feature enhancement of the image. CA provides location-aware information to help SK select the appropriate convolution kernel branches more accurately. At the same time, the multi-scale feature fusion ability of SK enriches the features processed by CA—the two form a complementarity.

This study is based on the YOLOv5 model. SIoULoss is used instead of CIoULoss, and NWDLoss is integrated into its loss calculation process. The SIOULoss can provide more accurate positioning guidance than the CIoULoss through angle perception and decomposition optimization capabilities. The NWDLoss further optimizes evaluating the box’s matching degree from the probability distribution perspective. The combination of the two reduces the positioning error. Meanwhile, the NWDLoss can still provide an effective gradient when the frames do not overlap, while the SIoULoss can perform better when the frames overlap. Combining the two improves the stability of the gradient during the training process.

Due to the invariance of the scale of NWDLoss, the sensitivity of the target scale is reduced through normalization processing, enabling the large and small targets to have a more equal position in the loss function. Combining the angle and shape optimization capabilities of SIoULoss, the SIoULoss function can effectively correct the influence caused by Angle deviation and better predict the aspect ratio of minor target defects. The combination of the two, supplemented by the feature expression of the attention mechanism, forms a complete small object detection solution from feature extraction to loss optimization.

This study utilizes two attention mechanisms during forward propagation to extract and fuse features in the neck network, suppress irrelevant backgrounds, and improve the quality of features. The loss function is used to predict errors in the subsequent training stage and guide the update of model parameters. It can be said that the attention mechanism provides more accurate eigenvalue guidance for the prediction error of the loss function and reduces its regression difficulty. The loss function guides the allocation of attention for the second round of the attention mechanism. Through continuous iterative calculations, the positioning accuracy of the model training is improved, the training stability is enhanced, and the generalization ability is enhanced.

## 3. Experiments and Discussion

### 3.1. Dataset

The training dataset used in this study only contains metal additive manufacturing defects of the type of holes. It consists of 1000 instances of hole defects, each annotated with precise bounding boxes. These bounding boxes provide comprehensive and detailed information about the target’s position, size, and category. In this study, 100 instances of hole defects were selected as the validation set for experimental verification.

### 3.2. Experimental Environment

This study’s experimental environment is the Windows 10 operating system. All experiments were conducted on the software platform Python-3.9.19 torch-2.2.2+cu121 CUDA:0 (NVIDIA GeForce RTX 3060 Laptop GPU, Santa Clara, CA, USA). The experimental parameters were set as follows: 100 epochs of training rounds. The batch size is 16, the input image size is 640 × 640, and all other settings are default.

### 3.3. Performance Index

This study adopted precision, recall, mean average precision (*mAP*), and regression loss as evaluation criteria to verify the advantages of the SCK-YOLOv5 model.

Herein, *n*: total number of categories; *Pij*: total number of pixels in the real image that belong to category *i* and are predicted as category *i*; ${ij}_{p}$: total number of pixels in the real image that belong to category *i* but are predicted as category *j*; *TP*: true-positive number, i.e., the number of pixels that are positive in the labels and also positive in the prediction values; *TN*: true-negative number, i.e., the number of pixels that are negative in the labels and also negative in the prediction values; *FP*: false-positive number, i.e., the number of pixels that are negative in the labels but positive in the prediction values; *FN*: false-negative number, i.e., the number of pixels that are positive in the labels but negative in the prediction values; *TP* + *TN* + *FP* + *FN* = total number of pixels; *TP* + *TN* = number of correctly classified pixels [28].

#### 3.3.1. Precision

Precision is also called inspection accuracy. It is the proportion of positive samples that are correctly predicted by the model. In defect detection, if the bounding box predicted by the model coincides with the real bounding box, it is considered a correct prediction.

The formula is as follows:

(18) $$Precision=\frac{TP}{TP+FP}$$

#### 3.3.2. Recall

The recall is also known as the detection rate. It measures the model’s performance in detecting. It represents the percentage of the correctly predicted positive samples among the actual positive samples. It is used to evaluate the model’s ability to identify all truly positive samples. If the actual bounding box coincides with the predicted bounding box, then this sample is considered to have been correctly recalled.

(19) $$Recall=Sensitivity=\frac{TP}{TP+FN}$$

#### 3.3.3. Average Precision

Average precision (*AP*) calculates the average precision across different categories. *AP* integrates the changes of precision and recall and is an essential metric for evaluating the advantages and disadvantages of defect detection models.

(20) $$\mathrm{AP}=\int_{0}^{1}p\left(r\right)dr$$

#### 3.3.4. *mAP* (Mean Average Precision)

*mAP* represents the average precision for multi-class problems. For binary classification problems, *mAP* = *AP*, and *mAP*50 indicates the *mAP* value at an *IoU* threshold of 50%. As a comprehensive indicator, it simultaneously reflects precision, recall, and mean average precision. The higher the *mAP* value, the more accurate the model is. The range of *mAP* values is [0, 1], and the closer it is to 1, the better the detection effect. *AP* and *mAP* are comprehensive evaluation indicators that reflect the algorithm’s accuracy in identifying targets for individual and all classes. Higher *AP* and *mAP* values indicate that the YOLO algorithm model has higher confidence in detecting target objects.

(21) $$\mathrm{mAP}=\frac{AP}{num_{c}lass}$$

#### 3.3.5. *mAP*50–95

*mAP*50–95 is a more stringent evaluation metric. It calculates the *mAP* values within the range of IoU thresholds from 50% to 95% and then takes the average. This enables a more accurate assessment of the model’s performance under different IoU thresholds.

#### 3.3.6. Regression Loss Class

Train/obj_loss is the loss value for objectness prediction during training. This pertains to the model’s ability to determine whether a specific object exists in an image. Val/obj_loss is the object loss value on the validation set, evaluating the model’s capability to detect objects in unseen data. Objectness loss is typically measured using binary cross-entropy (BCE) loss [28].

To evaluate the accuracy of the training model proposed in this study, many experiments were conducted for comparison in this paper. (1) Quantitative analysis was carried out to compare several popular YOLO detection algorithms. It was verified that compared with other YOLO models, SCK-YOLOv5 was optimized and improved based on the YOLO system algorithm, providing higher detection accuracy and faster speed. (2) Ablation experiments were conducted to determine the degree of influence of a condition or parameter on the results. Compared with the original YOLOv5, this paper has four new schemes or methods. In the ablation experiment, we controlled one condition and parameter at a time, and by observing how the results changed, we determined which condition or parameter had a more pronounced effect on the results. The comparative experiment compares the loss values of several well-known loss functions applied to the live network. This comparison highlights the differences between the model studied in this paper and other loss models. Regarding the generalization experiment, we aimed to verify that the high recognition accuracy of the model proposed in our study is not limited to the defect dataset collected in this study, and it can also be applied in other datasets. We performed generalization experiments by conducting comparative experiments on a large number of defect datasets downloaded from the Internet, through which the extensiveness of the YOLO model proposed in this study can be verified, and the technique is mainly used to evaluate the effectiveness of new theories or improvement measures.

### 3.4. Quantitative Analysis

**Figure 8:**
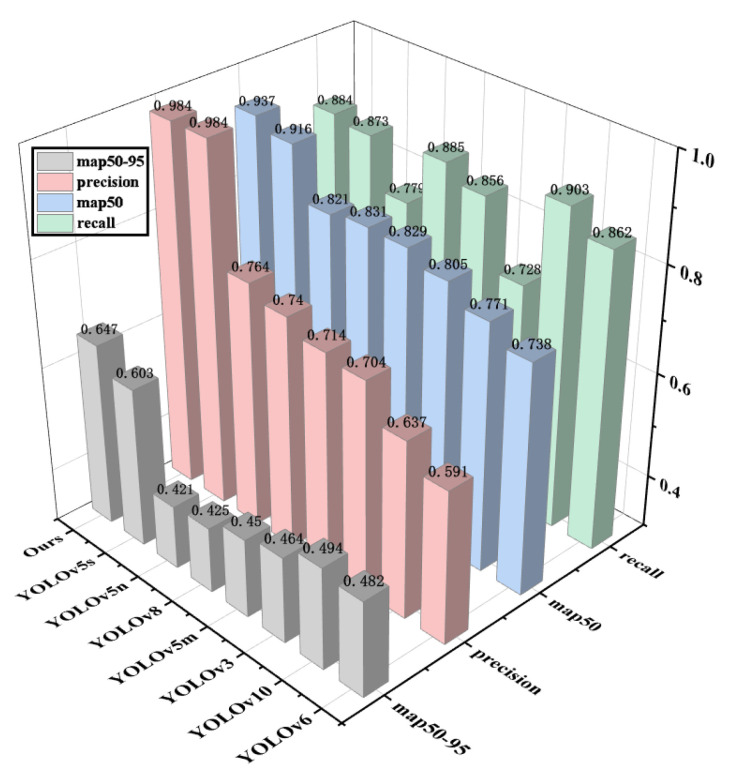
Three-dimensional comparison chart of three indicators of different YOLO models.

This study’s model was compared with commonly used methods and other improved approaches in recent years. In the quantitative analysis, the models selected by this study included YOLOv3, YOLOv5, YOLOv6, YOLOV8, YOLOv10, etc. The experimental results of different models are shown in Figure 1 and Figure 8.

From Table 1 of the experimental results, the research found relatively similar values in this comparative test. Random factors caused the difference in the indicator. To verify that the model itself truly improved, this study ran the model five times under the same conditions and calculated the mean of the precision rate, the mean of the recall rate, the mean of the *mAP*@50 rate, and the mean of the *mAP*@50–95 rate. It is observable that compared with other baseline models, the overall performance of the YOLOv5 model is superior. The accuracy of YOLOv3 is around 0.7, while the detection effects of YOLOv5m, YOLOv5n, YOLOv6, and YOLOv8 are relatively similar, with their detection accuracy reaching around 0.75–0.8. The recall rate of YOLOv10 is relatively high, reaching 0.903. Still, its precision rate and average precision rate are relatively low, at 0.637 and 0.771, respectively, which are 35.2% and 16.3% lower than the model in this study. The detection accuracy of YOLOv5s reaches 0.984; meanwhile, its recall rate and average precision rate reach 0.873 and 0.916, respectively, showing great superiority over other models, but compared with the model in this study, its precision average rate is 0.5% lower, average recall rate is 1.2% lower, and the *mAP*@50 is 1.8% lower. The map@50–95 metric of the model in this study improved by 4.4% compared to the original YOLOv5 model, and there was a significant improvement compared to other models in the YOLO series. The improvement of the map50-95 metric can prove that the introduction of SNWDLoss in this paper is of great help in improving the defect detection rate of small targets.

Through the intuitive comparison of the three-dimensional graph in Figure 8 based on the experimental results, it is verified that YOLOv3 and YOLOv4 are not as effective as YOLOv5 in visual recognition. Due to the imperfection of the code algorithm of YOLOv6-v10, it is inferior to YOLOv5 in recognizing small targets. Compared with the original YOLO algorithm without improvement, the overall performance of YOLOv5s is undoubtedly the best. Therefore, this research focuses on the improvement based on YOLOv5s. Its accuracy rate increased by 0.5%, the recall rate increased by 1.2%, and the correctness and coverage of the surface model also improved. At the same time, after the improvement, this research achieved an increase of 1.8% in the average precision rate. This indicates that the scheme of introducing CA and SK attention mechanisms and the SIoULoss function proposed in this research is correct.

**Figure 9:**
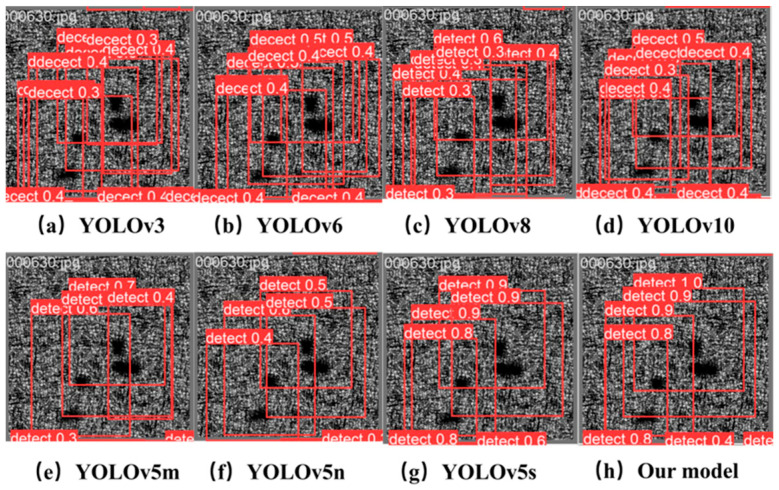
Actual detection results of surface defects by several optimal models.

To more intuitively prove that this study’s improved method is effective compared with other models, we selected several representative YOLO model detection cases for qualitative analysis. This study selected 630 specific representative images containing defects from the test set. As shown in Figure 9, they represent the prediction structures of the models in this study: YOLOv3, YOLOv4, YOLOv5, YOLOv8, YOLOv10, and SCK-YOLOv5.

Regarding the above algorithms, the recognition accuracy of YOLOv3 is mostly around 0.3, which is relatively low, and there are numerous false detections. The recognition accuracy of YOLOv6 improved to 0.4 compared to YOLOv3, but there are still countless false detections. The recognition accuracy of YOLOv5m and YOLOv5n is around 0.6, and although they identified all four defects without any missed detections or false detections, their recognition accuracy is still relatively poor. The recognition accuracy of YOLOv6 is around 0.5, and there are multiple false detections. Most of the recognition rates of YOLOv8 are around 0.4, and numerous false detections exist. The recognition rate of YOLOv10 is approximately 0.4 as well. Compared to YOLOv8, the false detection rate is lower. However, YOLOv5s has a relatively higher recognition rate, with an average accuracy of 0.9. Due to the other algorithms mentioned above, this research algorithm achieved a recognition result with a confidence level 1. It can identify all four defects in the image without any missed or false detections, demonstrating the superiority of the improved algorithm in this study and reflecting the flexibility and accuracy of the enhanced loss function.

### 3.5. Ablation Experiment

**Figure 10:**
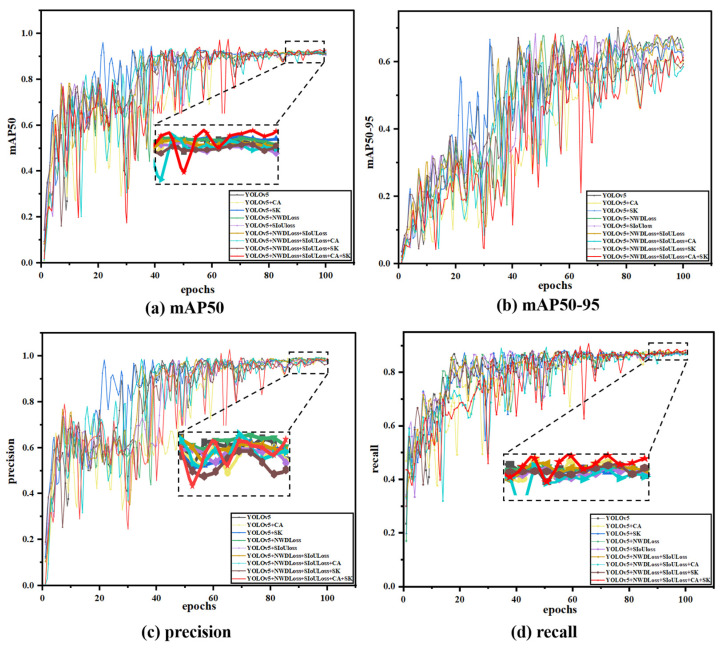
Comparison of ablation experimental results.

This study conducted ablation experiments to verify the improved model’s effectiveness. Ablation experiments are a scientific exploration tool that analyzes the significance of each variable on the final result by changing the key variables in the experimental design one by one. We can compare conditional, non-specific situations to identify the key parts of the system’s efficiency, and the advantage of this approach is that it provides evidence of causality, which we believe is necessary to compare the system’s efficiency. It helps us to study how each element affects the model’s performance, and we can add or remove individual elements one by one until we obtain the best performance of the model. In this study, we performed nine sets of improved ablation experiments, and the training results are shown in Figure 10, with different colored curves representing the training results of the model with various enhancement components.

Figure 9 shows the dynamic distribution of the gas cavity as the metal solidifies when the laser is completely stopped, and a gas cavity is formed in the middle of the melt pool. At this time, the laser radiation intensity is high, the depth of the small holes created during laser irradiation is appropriate, and the gas cavity is located in the middle of the metal material. When the laser stops heating, due to the gas cavity formation position and the distance between the metal surface being more appropriate, the gas cavity can reach the metal surface in a limited period. When the gas cavity reaches the metal surface, the liquid metal continues to solidify the molding. However, suppose the laser stops heating at an inappropriate time. In that case, it may result in the liquid phase metal not being able to fill all of the gas cavity when the gas cavity reaches the metal surface, resulting in an uneven metal surface.

As shown in Table 2, this study introduces four improvements to the YOLOv5 model to enhance its performance and efficiency. In this study, the code of YOLOv5s (a better-performing version of YOLOv5) was improved by introducing an attention mechanism and a loss function. It achieved specific improvements in accuracy, recall, and average accuracy. In this section, the results are analyzed through fusion experiments. Since several sets of results among the various indicators are pretty similar, and to avoid the lack of rigor in academic research due to numerical differences and fundamental differences, each group of experiments in this section was repeated for five training sessions, and then, the average value was taken, and the confidence range was marked.

The performance of precision and recall was relatively similar across the nine ablation experiments. After adding a single attention mechanism and loss function, both showed a certain degree of decline. The precision (P) decreased from 0.98 to 0.97, and the recall rate (R) dropped from approximately 0.873 to around 0.87. However, after integrating the two loss functions (SNWDLoss), the precision (P) rose to 0.987, which was higher than the original precision of 0.984. Subsequently, when integrating a single attention mechanism, the precision (P) decreased again, and in one of the groups, it was even lower than 0.712. After analysis, this might be due to the CA attention mechanism failing to extract the feature information processed by SIoULoss effectively. However, when combined with other improvement measures, the recall rate (R) improved to 0.878. Finally, after integrating four improvement measures and further optimizing the network structure, the precision (P) and recall rate (R) reached their maximum values of 0.989 and 0.885, respectively.

**Figure 11:**
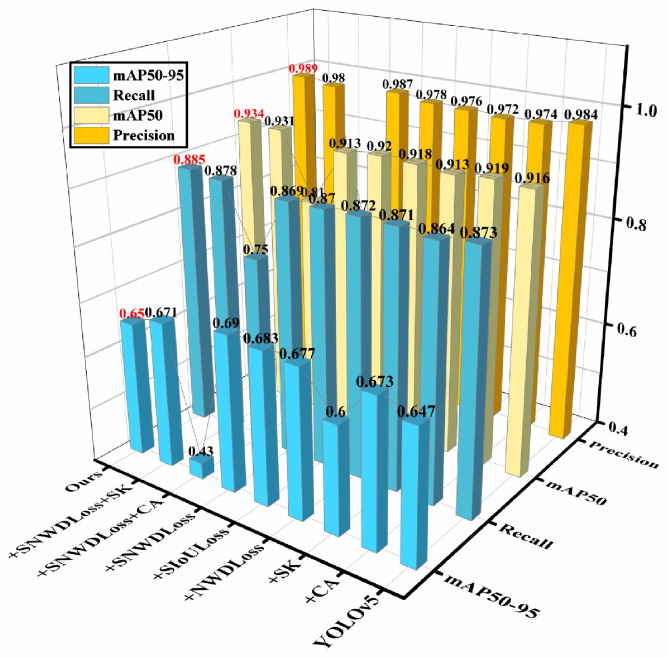
Three-dimensional comparison of ablation experiments.

The average precision (*mAP*) of the nine sets of ablation experiments can better reflect the model’s advantages in this study. After adding the attention mechanism and the loss function, the *mAP* improved to varying degrees, especially the CA attention mechanism, and the two loss functions has a significant enhancing effect on the *mAP*. After introducing CA alone and the two loss functions separately, the average precision increased to around 0.92. Then, the four improvements were fused, achieving an average accuracy of 0.934, 1.8% higher than before. Overall, the recognition effect was significantly improved. To facilitate a more intuitive comparison, this article presents the data in a three-dimensional format, as shown in Figure 11.

We analyzed the improvement mechanism. SIoULoss is a new-style loss function. In the training process using the classic cross-entropy loss on datasets with class imbalance, there are some problems. SIoULoss can solve these problems. It is particularly good at dealing with small objects and those that are hard to classify. In model training, we will encounter samples that are not easy to distinguish; for example, background noise is like some interference information, which will affect the judgment of the model. By adjusting the importance of negative samples, we pay more attention to non-interfering information. The model will not be affected too much by the interference information, such as background noise, and can concentrate more on learning those essential features, and the accuracy of the overall detection of the model will be improved. We introduced NWDLoss and SIoULoss to detect defects and segment instances so that the inspection results can be more accurate when encountering objects with complex shapes and significant size differences. Wasserstein distance is a more straightforward way to measure spatial differences in features. It is convenient to display the working process of the model visually. Coordinating attention mechanisms places more emphasis on location information. This allows the network to understand the position relationship between objects and the environment information they are in and measure the information more directly, which strengthens the perception of object boundaries and makes the model more accurate when locating the position of objects. Selective Kernel Attention allows the network to dynamically adjust the weights based on the local features of each pixel in the input image. Selecting and combining different filters makes the model more flexible when extracting features, and the extracted features are more targeted and unique. When we combine these four mechanisms in YOLOV5, we can effectively solve the problems of positioning accuracy, small target defect detection, and robustness of complex scenes in the defect detection process, making up for the shortcomings of the previous version, such as resolution dependence, false alarms, and missed detection. These mechanisms improve the model’s ability to understand the situation and process local details. Their combined effect is to optimize the model’s response to various conditions so that it can significantly improve defect detection performance while maintaining good speed.

**Figure 12:**
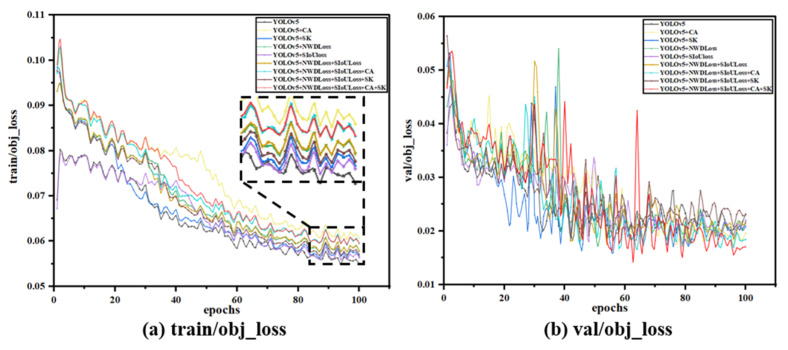
Loss regression plot at epochs = 100.

As shown in Figure 12, the validation loss (val_loss) of the model established in this study is significantly lower than that of other models, reaching a minimum loss value (close to 0.015). The lower the val_loss, the better the model’s understanding and fit of the validation data, the stronger the generalization ability, and the better the performance balance was achieved, and training strategies such as learning rate adjustment and regularization may have played a role in making the model less error-prone while maintaining consistency with new samples. Combined with these four mechanisms, YOLOV5 can effectively solve the problems of positioning accuracy, small target defect detection, and robustness of complex scenes in defect detection. It can compensate for possible defects such as resolution dependence, false alarms, and missed detections in the previous version. These improvements enhance the model’s global understanding and local detailing, and their combined effect optimizes the model’s response to various situations, allowing it to improve defect detection performance while maintaining speed significantly.

### 3.6. Loss Function Comparative Experiment

**Figure 13:**
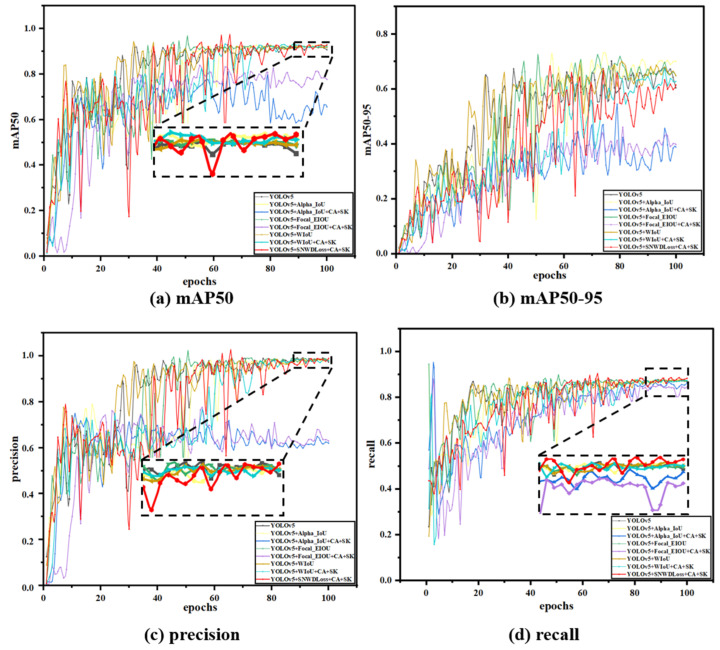
Comparison of the effect of the loss function.

To verify the advantages of the new loss function, SNWDLoss, proposed in this study, a comparative experiment was conducted. Several popularly improved loss functions, such as CIoU, WIoU, Focal_EIoU, and Alpha_IoU, were compared with the model of this study. Different loss function models are shown in Figure 13.

SNWDLoss is an innovative loss function composed of SioULoss and NWDLoss. Compared with other loss function models, the newly proposed SNWDLoss has greatly improved accuracy, recall, and average accuracy. Unlike traditional IOU losses, this study’s design takes into account both the accuracy of defect edge detection and the consistency of the overall structure.

In Figure 13, this study found that SNWDLoss exhibited higher accuracy and completeness in the target recognition task, and its accuracy (P), recall (R), and average accuracy (*mAP*) achieved the best performance among the above models. This may be because SNWDLoss considers both center point and boundary information, improving the accuracy of object positioning, reducing misclassification and missed detections, and helping to alleviate confusion between classes, as SNWDLoss is partly concerned with dice coefficients. SNWDLoss exhibits higher robustness and generalization than loss functions focusing only on a single factor.

Thanks to the better boundary-matching mechanism provided by SIOU, it can handle a variety of complex scenarios, including small target defect detection and background interference, and the experimental results confirm that SNWDLoss, as a new type of loss function, has a significant effect on improving the overall performance and stability of the model. This also shows that the design strategy of the loss function can effectively enhance the performance of the deep learning model in the field of defect detection. The advantage of SNWDLoss lies in its comprehensive and targeted optimization, which enables the model to make more accurate predictions in defect detection tasks and better demonstrate performance benefits in real-world applications.

### 3.7. Universal Experiment

To demonstrate the advantages of the current research model compared to other models, this study conducted a qualitative analysis of the SCK-YOLOv5 and YOLOv5 models. A steel surface defect dataset (NEU-DET) created by the Song Kechen team from Northeastern University was selected. This dataset contains 1800 images. The images in this dataset are widely used by many people and have not been processed before. This universal experimental study did not perform any image processing-related operations on the images within the NEU-DET dataset. The first column shows the test results of the YOLOv5 model, and the second column shows the test results of the KSC-YOLOv5 model.

**Figure 14:**
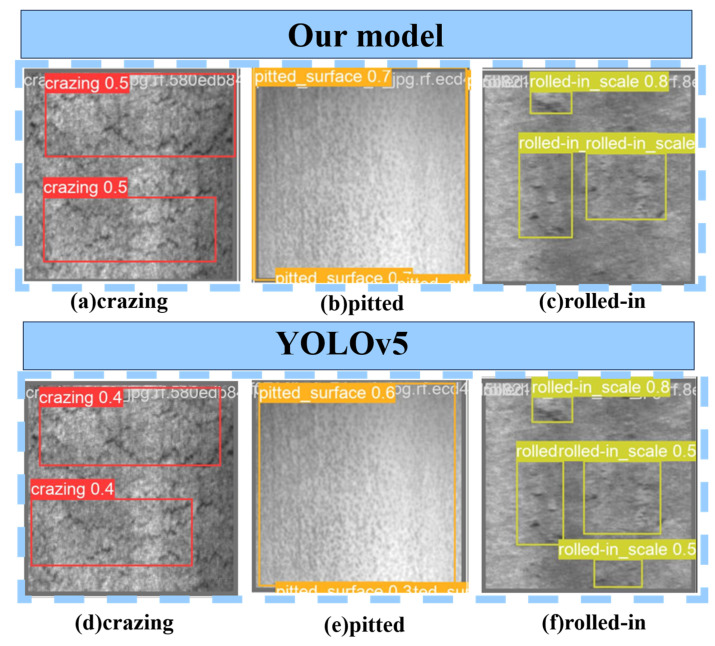
Comparison chart of the visual recognition effect of the universal experiment.

The results in the graph in Figure 14 illustrate how well the new model proposed in this study performs in dealing with missed and false detections. Compared with the YOLOV5 model, which already has relatively good recognition accuracy, the SCK-YOLOV5 model in this study shows higher accuracy in detecting metal surface defects such as cracks and pitting surfaces. The SCK-YOLOV5 model makes up for the missed detection of YOLOV5 in rolling in-scale inspection, effectively reducing the error rate. These image representations emphasize the SCK-YOLOV5 model’s ability to accurately identify metal surface defects even in complex visual scenarios, thereby enhancing the robustness and reliability of visual recognition in real-world applications. As shown in Figure 14.

**Figure 15:**
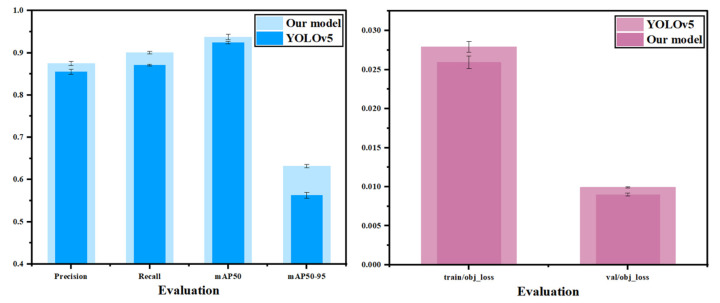
Comparison chart of universal experimental data.

To further verify the numerical performance of the model in this study on the NEU-DET dataset, bar chart data comparison is adopted such Figure 15 in this section. The mean values of the five experiments in each group were taken. The black intervals in the figure represent the confidence intervals of each experimental index. It can be seen as shown in the figure that in the four metrics of precision (P), recall (R) rate, *mAP*@50, and *mAP*@50–95, the model studied in this experiment is significantly superior to the source YOLOv5 model, and there is no overlap of confidence intervals between the models, which proves the practicability of the model studied in this experiment on the NEU-DET dataset.

## 4. Conclusions

Precisely detecting surface defects in metal components made through additive manufacturing poses substantial technical challenges. One of the thorny issues is dealing with highly reflective surfaces. Such surfaces can cause traditional optical measurement methods to yield distorted results. As industrial applications demand micron-level quality control for crucial components more often, the existing vision systems are struggling. They have trouble maintaining the accuracy of inspections when faced with different degrees of surface reflectivity and complex defect shapes. To tackle these limitations, we studied and developed the SCK-YOLOV5 framework.

We created this framework by systematically integrating deep learning optimization techniques and the principles of polarization physics. Our proposed architecture combines the SIoULoss with the normalized Wasserstein distance (NWD) metric learning. This combination is beneficial as it helps enhance the stability of the bounding box used for object detection. Additionally, it boosts the system’s sensitivity to minor defects that might otherwise be overlooked. We employed a hybrid attention mechanism that utilizes coordinate attention (CA) and selective kernel (SK) convolution. This mechanism enables the system to extract features adaptively across various spatial scales. Moreover, the framework can effectively reduce specular reflection by integrating polarization imaging. In doing so, it can retain the defect features that traditional RGB-based systems usually fail to capture or tend to obscure.

Experimental verification has demonstrated that our SCK-YOLOV5 framework significantly outperforms the baseline YOLOV5. Specifically, the accuracy increased by 0.5 percentage points, the recall rate increased by 1.2 percentage points, and the *mAP*@50 improved by 1.8%. Our framework provides a reliable method for automatically detecting defects in high-precision additive manufacturing applications. Also, it successfully bridges an essential gap between the field of surface physics and data-driven inspection algorithms. However, SCK-YOLOv5 still has room to improve its accuracy of defect recognition. To further optimize the model, we plan to apply it to datasets other than defect detection.

## Figures and Tables

**Table 1:** Quantitative comparison of experimental results of the YOLO model.

Model	Precision	Recall	*mAP*50	*mAP*50–95
YOLOv3	0.704	0.728	0.805	0.464
YOLOv5s	0.984 ± 0.002	0.872 ± 0.003	0.917 ± 0.001	0.603 ± 0.004
YOLOv5m	0.714	0.856	0.829	0.450
YOLOv5n	0.764	0.779	0.821	0.421
YOLOv6	0.591	0.862	0.738	0.482
YOLOv8	0.740	0.885	0.831	0.425
YOLOv10	0.637	**0.903**	0.771	0.494
Our model	**0.989 ± 0.003**	0.885±0.005	**0.934 ± 0.002**	**0.647 ± 0.002**

**Table 2:** Ablation experiments test the results of the experiment.

Model	Precision	Recall	*mAP*50	*mAP*50–95
YOLOv5	0.983 ± 0.002	0.873 ± 0.003	0.916 ± 0.001	0.647 ± 0.004
+CA	0.976 ± 0.003	0.864 ± 0.009	0.919 ± 0.003	0.673 ± 0.006
+SK	0.971 ± 0.006	0.871 ± 0.007	0.914 ± 0.006	0.601 ± 0.004
+NWDLoss	0.972 ± 0.004	0.873 ± 0.002	0.918 ± 0.009	0.677 ± 0.009
+SIoULoss	0.973 ± 0.007	0.870 ± 0.006	0.920 ± 0.005	0.683 ± 0.002
+SIoU+NWDLoss(SNWDLoss)	0.987 ± 0.002	0.867 ± 0.012	0.912 ± 0.010	**0.688** ± 0.004
+NWDLoss+SIoULoss+CA	0.967 ± 0.009	0.869 ± 0.005	0.911 ± 0.010	0.572 ± 0.005
+NWDLoss+SIoULoss+SK	0.980 ± 0.003	0.878 ± 0.002	0.931 ± 0.001	0.671 ± 0.005
+NWDLoss+SIoULoss+CA+SK	**0.989** ± 0.003	**0.885** ± 0.005	**0.934** ± 0.002	0.650 ± 0.002

## Data Availability

The original contributions presented in this study are included in the article. Further inquiries can be directed to the corresponding author.
